# Can Training Make Three Arms Better Than Two Heads for Trimanual Coordination?

**DOI:** 10.1109/OJEMB.2023.3305808

**Published:** 2023-08-16

**Authors:** Yanpei Huang, Jonathan Eden, Ekaterina Ivanova, Etienne Burdet

**Affiliations:** ^1^ Department of BioengineeringImperial College of Science Technology, Medicine 4615 SW7 2BX London U.K.; ^2^ Department of Mechanical EngineeringUniversity of Melbourne 604653 Parkville VIC 3052 Australia; ^3^ School of Electronic Engineering, Computer ScienceQueen Mary University of London 4617 E1 4NS London U.K.

**Keywords:** Foot control, teleoperation, three-hand surgery, Tri-manipulation

## Abstract

Supernumerary effectors have been proposed to enable users to perform tasks alone that normally require assistance from a partner. While various supernumerary robotic limbs have been developed in the last decade, the capability of users to operate them effectively has not yet been proven. Here we tested whether users (i) can complete a task that requires simultaneous and fine control of three effectors, and (ii) can be trained to do so with similar or superior performance as through collaboration with a human partner. As in previous studies, initial augmented capability was less than that of working with a partner. However, one hour of dedicated solo trimanual training across three days significantly increased task performance, so that participants became able to perform trimanual control alone as well as or better than they could with a new partner. This shows the viability of augmentation systems for applications such as in robotic surgery or industrial assembly, which can be further validated on real tasks with physical systems.

## I. Introduction

Degree of freedom (DoF) augmentation with supernumerary effectors (SEs) aims to extend the DoFs available to a user for a given task [1]. If effective, this could enable an individual to carry out alone tasks requiring more than two hands, such as fastening a board to an overhead assembly or stitching two nerves together during surgical anastomosis. Despite various robotic SEs having been developed [2] and shown suitable for tasks such as industrial processes [3], assistive technology [4] and robotic surgery [5], their usefulness and efficiency have not yet been tested systematically. Furthermore, the tasks that have been investigated typically could be performed with just the two natural hands [6], [7]. *To be adopted, augmentation with a SE should (i) enable users to complete tasks that require more than two hands successfully, and (ii) do so with similar or superior performance as through collaboration with a human partner*.

For the successful completion of tasks, trimanual coordination has been implemented using autonomous augmentation [3], [8] of the third limb or manual control [6], [7]. However, in unstructured and variable environments such as in surgical applications, the responsibility for safety and ethics will rest with the human operator. Furthermore, complex or novel interventions will require direct control. Therefore, it is important to continue developing better control strategies for surgeons, such as possibly trimanipulation.

When manual control has been considered, initial evaluation of trimanual coordination with a SE has focused on examining whether users can perform tasks with more than two ‘hands’ using other body segments not required for the task [5], [6], [7], [9]. Here, it has been shown that using an additional ‘hand’ can be more efficient than using the two natural hands for control [5], [6], [7], and that users can extend from bimanual to trimanual tasks with limited loss of performance [9]. However, in a recent single-day study comparing solo trimanual operation to working with a human partner, the solo operation was less preferred and had inferior performance across tasks with different amounts of coupling between the hands [10]. While these results may indicate that augmentation cannot fulfil the above conditions, the observed differences may rather reflect insufficient training in the control of SEs. Here, previous studies have not provided dedicated training time as has been shown necessary for learning new augmentation devices [11] or skills [12].

When a pair performs a task they are able to draw upon a lifetime of interactions working with other people. For augmentation, there is typically no experience of controlling extra DoFs and users must instead rely on the transfer of existing natural skills [1]. It has been shown that bimanual behaviours cannot necessarily be built from unimanual actions [13], [14]. Similarly, initial comparisons of trimanual performance may suffer from participants lacking knowledge of how to perform tasks trimanually and instead trying to build from unimanual and bimanual behaviours. It is known that motor learning derived through dedicated training can improve user performance in new skills [15] and enable the learning of modified visuomotor mappings [16]. Motor learning has also been observed in augmentation experiments [10], [11], [17].

Since solo trimanual coordination with a given system needs to be trained only once, as opposed to learning to work with a partner that needs to be repeated with each new partner [18], we ask *can solo trimanual performance become as good or better than when working with a human partner through training of trimanual coordination?* To investigate this question, we designed a paradigm in which participants trained over three days in a robotic surgery inspired task from [10] requiring fine, coordinated control of the three effectors, and analyzed the performance, perception and coordination changes.

## II. Results

**Figure 1.**
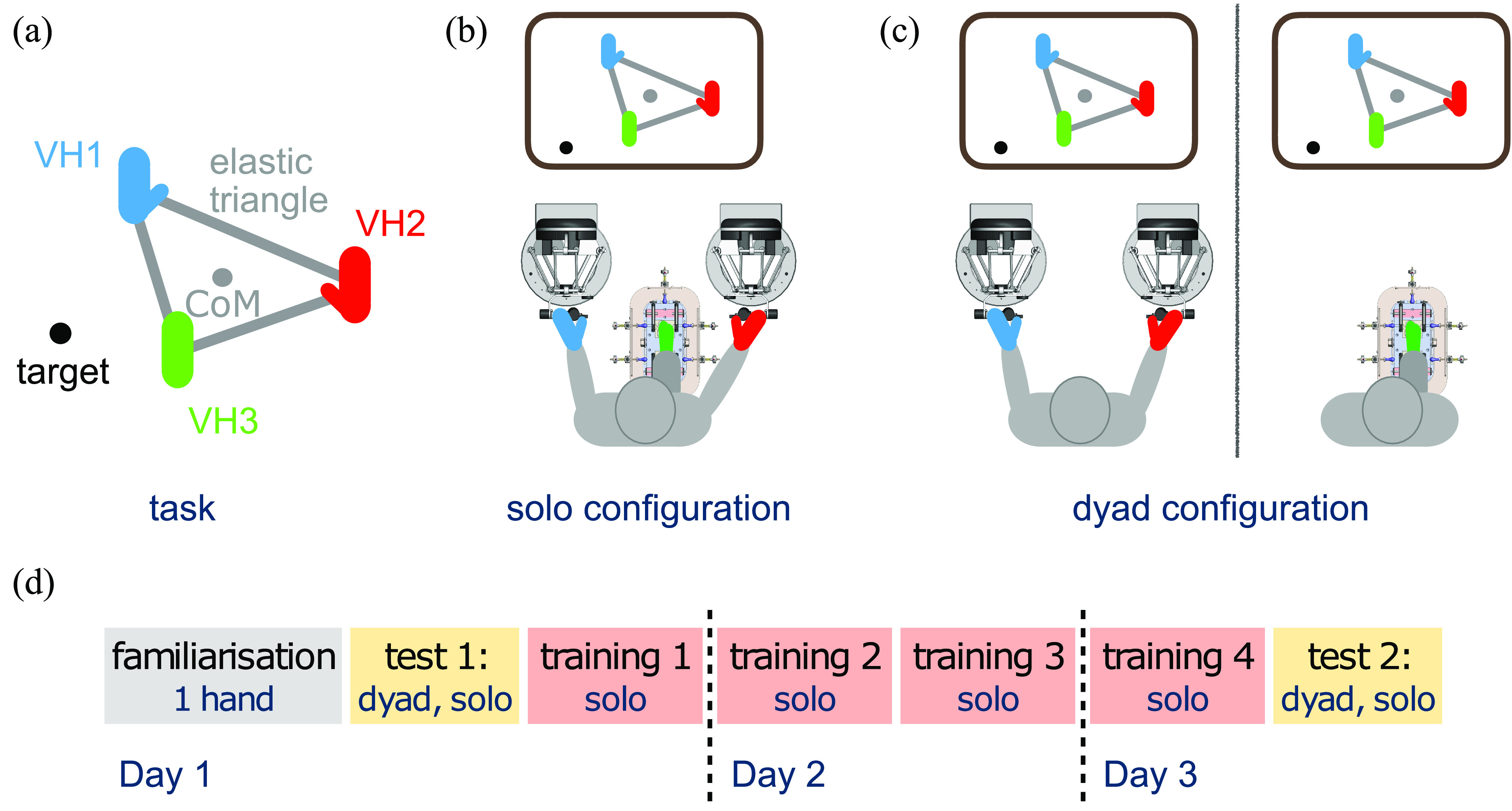
Comparison of solo trimanual and dyad operation of a task requiring simultaneous fine control of three virtual hands (VHs). (a) Participants had to bring the center of mass (CoM) of a triangle to the target by moving its vertices commanded by the hands and one foot while maintaining its shape. (b) In solo mode, the participant controls the hands and foot interfaces simultaneously. (c) In dyad mode, the two participants are separated by a curtain, one operating the hands interfaces and the other the foot interface. (d) The three-day experiment protocol consisting of four solo training repetitions and two tests of both solo and dyad configurations.

Three *virtual hands (VHs)* were controlled by two hand interfaces and a foot interface, and connected to the respective vertices of a virtual elastic triangle. The task required the triangle's center of mass (CoM) to reach a series of targets without losing its shape (Fig. 1(a), see Materials and Methods). 16 participants performed the task in two *configurations* (see Supplementary Materials, for example motion trajectories and speed profiles): *solo*, where they controlled the VHs using all interfaces (Fig. 1(b)); or as a *dyad*, in which pairs of participants separated by a curtain controlled the VHs, one using the hand interfaces and the other the foot interface with their preferred foot (Fig. 1(c)). The tracking performance was initially tested in both configurations, then solo performance was trained over three days consisting of one training repetition on the first day, two on the second and a final repetition on the third day (Fig. 1(d)). After training, an additional test was conducted and comparison of solo and dyad performance was undertaken through objective metrics and a user questionnaire.

### A. Solo Trimanual Performance Improved With Training

**Figure 2.**
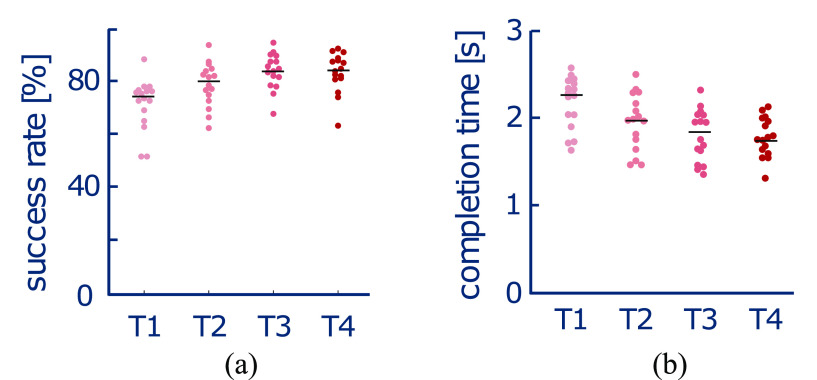
Change in solo performance for success rate (a) and completion time (b) for each participant with learning, where T1-T4 represent Training Repetitions 1 to 4 as in the protocol shown in Fig. 1(d). Note that each dot represents a single participant and the black horizontal line denotes the median of all participants.

Performance was evaluated through the percentage of trials for which the participants could reach the target before it refreshed every 3 s (*success rate*) and the time that it took to reach the target (*completion time*). All participants were able to perform the task, with their success rate (Fig. 2(a), Friedman test: $\chi ^{2}(3)=29,\,p< 0.0001$) and the average completion time for reaching each target (Fig. 2(b), one-way repeated measures Analysis of Variance (rmANOVA): $F(3,45)=37.58,\,p< 0.0001$) changing with training. There was a clear performance improvement over Training Repetitions 1 and 2 (success rate: $Z=-3.2329,\,W=5.5,\,p=0.0011$; completion time: $t(15)=5.127,\,p=0.0002$) and Repetitions 2 and 3 (success rate: $Z=-3.0539,\,W=9,\,p=0.0019$; completion time: $t(15)=5.483,\,p=0.0002$). Performance then stabilised between Repetitions 3 and 4 (success rate: $Z=0.0517,\,W=69,\,p=0.9699$; completion time: $t(15)=0.517,\,p=0.6125$). These results indicate that participants improved their ability for solo trimanual coordination and learned to successfully incorporate the ‘third hand’. As the performance stabilised by the final training repetition, the subsequent analysis only compares the differences between the trained behaviour (Testing Phase Day 3) and the initial performance (Testing Phase Day 1).

### B. Solo Trimanual Performance was as Good as in Dyads After Training

**Figure 3.**
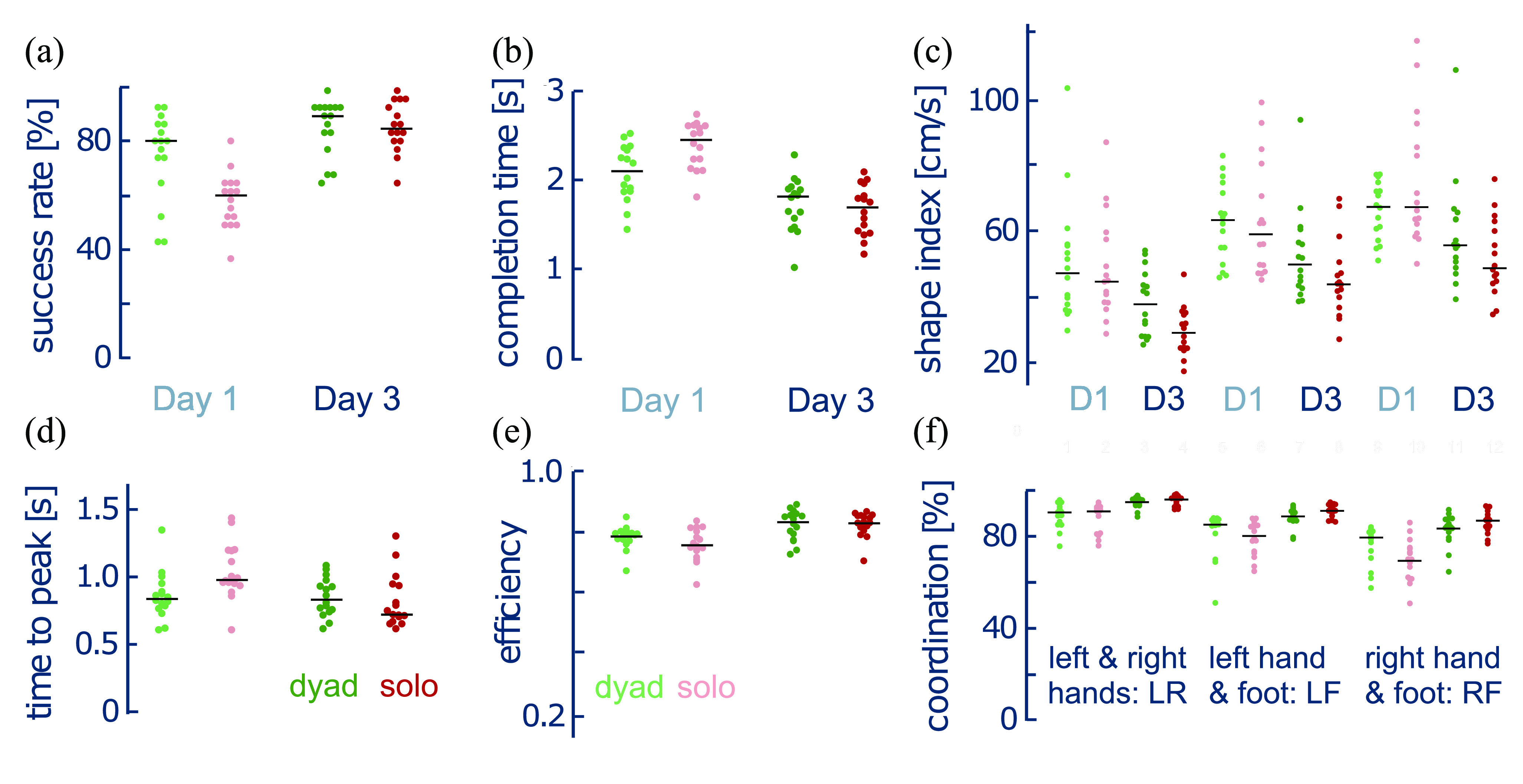
Performance in dyad vs. solo trimanipulation. The panels show the success rate (a), completion time (b), time to peak (d), and motion efficiency ratio (e) for different days, as well as the shape index (c) and coordination rate (f) for different hand combinations, where D1 and D3 are short for Day 1 and Day 3. Note that each dot represents a single participant and/or dyad configuration and the black horizontal line denotes the median of all participants.

In the initial test on Day 1, the dyad configuration possessed a higher success rate (Fig. 3(a), $Z = 2.7416,\,W = 96,\,p = 0.0128$) and a shorter completion time (Fig. 3(b), $t(45) = 4.140,\,p = 0.0003$) than the solo trimanual configuration. However, by the test on Day 3, participants exhibited similar performance in the solo and dyad configurations ($Z = 0.2206,\,W = 56,\,p = 0.8379$), where in both cases they were able to successfully complete the task more than 80% of the time, and had a similar completion time ($t(45) = -0.924,\,p = 0.3603$). This indicates that with solo configuration training, solo trimanual reaching becomes as good as when working as a dyad. Interestingly, despite only training in the solo configuration, the dyad configuration performance also improved for each of the task performance metrics from Day 1 to Day 3 (success rate: $Z = -2.497,\,W = 10,\,p = 0.020$; completion time: $t(45) = 4.466,\,p = 0.0002$).

The participants' ability to preserve the triangular shape was evaluated through the average summed deviation of the triangle side lengths (*shape index*), where the individual spring deviations are depicted in Fig. 3(c). Participants showed no difference between configuration for the shape index (configuration main effect: $F((1,45)=1.9425,\,p = 0.170$, configuration-test interaction: $F((1,45)=1.9326,\,p = 0.171$), however, there was an effect of the session ($F((1,45)=48.0459,\,p < 0.0001$) in which the shape index decreased from Day 1 to Day 3. This shows that while training improved the participant's ability to preserve the shape, this improvement occurred for both configurations.

### C. Coordination Evolved Differently for Different Configurations

The participants' level of coordination throughout the reaching motion was evaluated through the percentage of time in which different combinations of the two VHs moved concurrently (*coordination rate*). The difference in coordination between tests depended on the configuration (Fig. 3(f), two-way Aligned Rank Transform (ART) rmANOVA: $F(1,75) = 21.5135,\,p < 0.0001$), hand combination ($F(2,75) = 6.4089,\,p = 0.0027$) and their interaction ($F(2,75) = 4.0685,\,p = 0.0210$). The dyad configuration showed uniform improvement for all hand combinations (LF-RF: $Z = -0.5171,\,W = 58,\,p = 1.0$; LF-LR: $Z = 0.8791,\,W = 85,\,p=1.0$; RF-LR: $Z = 0.8273,\,W = 84,\,p = 1.0$). In contrast, the trimanual configuration showed greater improvement in combinations involving the foot-controlled hand compared to the dyad (LF – solo vs. dyad: $Z = 2.2235,\,W = 111,\,p = 0.1248$; RF – solo vs. dyad: $Z = 2.9474,\,W = 125,\,p = 0.0134$; RL – solo vs. dyad: $Z = 1.3444,\,W = 94,\,p = 0.7712$). This resulted in differences in the improvement across hand combinations, where the LF combination improved more than the LR combination ($Z = 2.5854,\,W = 118,\,p = 0.0534$) as well as the RF combination improved more than the LF ($Z = -2.4303,\,W = 21,\,p = 0.0786$) and LR combinations ($Z = 3.5162,\,W = 136,\,p = 0.0003$). These results indicate that while coordination improved across both configurations, the largest improvement derived from the solo configuration foot-hand coordination.

To further understand the participant behaviour, the *time to peak* speed was also computed across the testing phase (Fig. 3(d)). Interestingly, while there was no clear difference between the solo and dyad configurations for Day 1 ($Z = -2.1201,\,W = 27,\,p = 0.1006$) or Day 3 ($Z = 0.56899,\,W = 79,\,p = 1.0$), improvement across tests was only observed in the solo configuration ($Z = 3.1806,\,W = 129.5,\,p = 0.0018$).

### D. Training Resulted in Similar Task Perception

**Figure 4.**
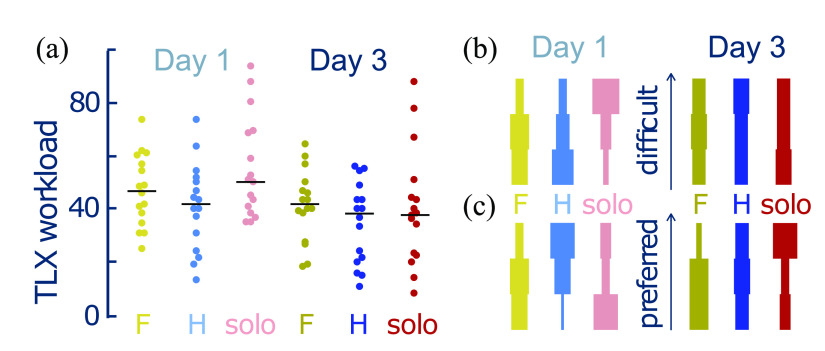
Perception of workload for the different configurations. (a) Workload (NASA TLX score). How operators perceived the (b) “difficulty” and (c) “preference” to carry out trimanipulation “solo” or by operating the foot interface “F” or the two hand interfaces “H” in the dyad configuration. Note that (b) and (c) depict discrete violin plots in which the bar width represents the percentage of participants for a given response.

The participant's perception of workload and their relative preference and difficulty was evaluated after each test phase through the raw NASA Task Load Index (TLX) [19] and by ranking the preference/difficulty for each role that the participant could be assigned {dyad configuration controlling the foot, dyad configuration controlling the hands, solo configuration}. The workload perception (Fig. 4(a)) changed between tests (Two-way rmANOVA: $F(1,15) = 23.1092,\,p = 0.0002$) and the interaction between the test and condition ($F(2,30) = 4.2270,\,p = 0.0241$). Similar to the task performance, there was initially a difference between the perceived workload in the solo and two hands dyad conditions ($t(15) = 3.907,\,p = 0.0112$). However, training reduced the perceived solo workload ($t(15) = 4.849,\,p = 0.0019$), so that there was no longer any clear difference by the Day 3 test ($t(15) = 0.959,\,p = 0.7383$).

The ranked difficulty (Fig. 4(b)) and preference (Fig. 4(c)) showed similar trends, in which the ranking was on Day 1 impacted by the configuration (Friedman test: $\chi ^{2}(2) = 6.125,\,p = 0.0468$ and $\chi ^{2}(2) = 6.125,\,p = 0.0468$, respectively) before no difference in the ranking was observed on Day 3 ($\chi ^{2}(2) = 0.125,\,p = 0.9394$ and $\chi ^{2}(2) = 3.125,\,p = 0.2096$). While post-hoc analysis did not reveal a clear preference for any condition, the trend suggested that the solo configuration was initially perceived as the most difficult and least preferred before the perceived difficulty became equivalent and the solo configuration was preferred after training. These results suggest that analogous to task performance, training reduced the perceived workload associated with solo trimanual operation, such that it became similarly perceived to that of the dyad.

## III. Discussion

*Can solo trimanual performance become as good or better than when working with a human partner through training of trimanual coordination?* Our results show that despite dyads initially outperforming solo trimanual operation, as in the coupled task first used in [10], one hour of solo trimanual training over three days is sufficient to reach a similar performance level. While working with a partner enables the potential for parallel planning and action, it also introduces uncertainty, whereby the operator must understand their partner's intentions and co-adapt in the event that this changes for either member of the partnership [20]. For the tested task which requires the continuous sharing of information between the hands, the results indicate that training is sufficient to reduce the negative effect of the increased workload while benefiting from the user having complete knowledge of each ‘hand's’ actions. Continuous information sharing is however not common to all trimanual scenarios such that further testing in other tasks is needed.

*How did participants appreciably increase their performance during solo trimanipulation training?* To understand how this improvement could be explained by altered movement behaviour, we analysed *motion efficiency* through the ratio of the CoM optimal travel distance against the actual travelled distance (Fig. 3(e)). This metric increased across the days (two-way ART rmANOVA: $F(1,45) = 30.2341,\,p< 0.0001$), suggesting that the participants changed their behaviour to plan more direct CoM motion. However similar to the shape index and time to peak, the motion efficiency was not affected by the configuration (main effect of condition: $F(1,45) = 0.8843,\,p = 0.3520$, interaction between condition and day: $F(1,45) = 0.8838,\,p = 0.3522$). Therefore while some improvement in task performance can be derived from a more efficient motion control of the CoM, the greater improvement in the solo configuration cannot be ascribed only to altered movement characteristics.

One possible explanation for the different improvements across configurations comes from the coordination rate. As expected participants moved their two natural hands with a high degree of coordination, which remained high and similar throughout. In contrast, in the solo configuration, the coordination between either the natural hand and the foot-controlled hand initially had a lower coordination rate before a large improvement between the two testing periods. This suggests that while participants might have moved their limbs equally efficiently in the solo or dyad configuration, the training improved the solo participant's ability to coordinate their foot's movement with the other limbs such that they could execute the task more effectively. This indicates that most performance increase stemmed from improved foot-hands coordination acquired with training. It has been previously observed that ipsilateral hand-foot coordination can be worse than that of the contralateral case during anti-phase motion [21]. Our findings suggest a similar result for in phase motion that might have acted as a limit to previous trimanual coordination studies [10], that training was able to improve.

Interestingly, training of the task in the solo configuration also resulted in improved performance for participants when working in the dyad configuration. While this improvement may partially result from the learning that took place from the dyad's previous testing phase working together, and suggests some potential interference that may have occurred due to the participants seeing both configurations in the initial test, it does indicate that some of the improvement may be derived from transferable skills such as the planning of the CoM's motion or the coordination between the limbs. This indicates that some skills necessary for working in a team, such as in the collaboration between a surgeon and their assistant, might be trained alone. It also suggests the potential for further dyadic improvement with additional training such that our results may change when compared to long-term (trained) working relationships. However, while an individual would only need to learn to perform three-handed tasks once, they would need to learn each new working relationship (as is known from teamwork in surgery [18]), thereby limiting potential efficiency.

## IV. Conclusion

Successful augmentation with a SE should allow users to complete tasks that require more than two hands with similar or superior performance as when working with a human partner. While autonomous SE operation can reduce the operator workload when completing a task, for safety and ethical reasons the operation of a human is still essential. Therefore, it is important to continue developing better control strategies for surgeons, such as trimanipulation.

Here we tested the difference in performance and perception between individuals manually operating trimanually and them working with a fixed partner both before and after dedicated solo trimanual training. Similar to previous findings [10], the participants of our experiments initially preferred carrying out trimanipulation in a dyad and exhibited better performance in this configuration. However, this drastically changed with dedicated trimanual training in the solo configuration. In four 15-minute repetitions across three days, the participants increased task performance relative to their initial solo and dyad levels. After this training, all perception and performance measures became similar between the dyad and solo configurations.

The results of this study extend previous results that showed that trimanual performance can exceed bimanual performance in independent tasks [6], [7], showing for the first time that training alone is sufficient to remove the initially observed differences between dyadic and solo performance [10]. This suggests that trimanipulation with a SE fulfils the basic requirements for augmentation adoption, although this needs to be validated on real world task with a physical SE.

## V. Materials and Methods

### A. Participants

The experiment was approved by the Research Governance and Integrity Team at Imperial College London (Reference: 21IC6935). 16 participants (six female, ten male) without known motor impairment aged 27.6 $\pm$ 4.4 years participated in the experiment. 14 participants were right-handed (Edinburgh handedness inventory score $> $ 60) with the remaining two were inconsistent handers favouring their right hand [22], while 15 participants were right-footed according to the ball-kick dominant leg test [23]. All participants provided their written informed consent before starting the experiment.

### B. Experimental Design

The experimental task was designed to simulate everyday activities in which continuous knowledge of the three ‘hand's’ position is required to physically coordinate their motion, e.g. holding a tray or operating on a common tissue in surgical anastomosis, and considered the same ‘3-coupled’ task as [10]. It was conducted in virtual reality to control for the effect of different visualisations and to ensure consistency with [6], [9]. The participant/s controlled the vertices of an elastic triangle and were asked to reach for a target with the triangle's CoM, while preserving its initial equilateral shape and size (Fig. 1(a)). The target's position was updated to randomly move by a fixed distance in one of eight set target directions on the screen every three seconds.

The setup consisted of visual displays, two hand interfaces with gravity compensation (Omega7, Force Dimension), and one custom-built foot interface which provided continuous support to minimise fatigue [24] (Fig. 1(b), (c)). Participants were instructed to face their respective (horizontally placed) monitor which displayed visual feedback of their performance. Three *virtual hands* (VHs) were displayed (Fig. 1(a)), where the blue VH1 was controlled by the left hand, the red VH2 by the right hand and the green VH3 by the foot interface. The horizontal planar movement of each interface was then recorded and mapped to the horizontal planar screen position of the respective virtual hand at 30 Hz frequency.

Participants performed the task in two different configurations. In the *solo* configuration (Fig. 1(b)), one operator controlled the two hand interfaces and the one foot interface. In the *dyad* configuration (Fig. 1(c)), two operators collaborated to perform the task, where the hand interfaces were controlled by one operator and the foot interface by the other.

Participants received visual feedback if they successfully reached the target by its color changing from black to green. Additionally, consistent with [9], [10] they received feedback of their preservation of the reference shape through visual feedback. When the distance between any two VHs was too large (i.e. more than 20% larger than the original length), the triangle edge connecting these two VHs thinned. With further increases this thinning continued until the edge ultimately disappeared if the distance became more than 30% larger than the original length (suggesting that the elastic had been broken); when the distance of two VHs was too close (i.e. more than 20% smaller than its original length), the edge widened and would ultimately show a collision mark if the distance became smaller than the original length by more than 30% (suggesting that the triangle collapsed). A video of the experiment is attached with a single participant in the solo configuration (https://youtu.be/z79_UQnD-ag). No haptic feedback was provided by the interfaces.

### C. Protocol

The three-day experiment took place over consecutive days and combined the familiarization, testing and training phases (Fig. 1(d)). On Day 1, the participants first conducted the familiarization phase to gain experience using the interfaces and understand the mapping between the interface position and monitor coordinates. They then conducted baseline testing of their abilities in both the dyad and solo configurations before finishing the Day 1 session by carrying out the first repetition of the solo training phase. On Day 2, the participants continued training with two further repetitions of the solo training phase with a short break between the two repetitions. On Day 3 they carried out a fourth training phase repetition, followed by a final testing phase (with the same partner) to assess their dyadic and solo trimanual capability.

In the *familiarization phase*, the participant/s used their respective interface to control each VH in a reaching task. As in the main task, a single target was shown on the screen and its position was updated every 3 s. Each participant was asked to reach for the randomly appearing target with the indicated VH for 32 targets per VH.

In the *testing phase*, the participant's ability to perform the main task was assessed in both the solo trimanipulation and dyad configurations, where participants remained in the same dyad for both testing phases. The participants conducted blocks of 32 reaching trials for each configuration. In the solo configuration, only one block was performed for each test, while in the dyad configuration participants took the two possible roles and therefore conducted two blocks. The order of blocks was randomised such that half of the participants started in the solo configuration and the other half in the dyad configuration. In addition, the testing phase considered a bimanual model (which was not otherwise used in the study). Here, the participants performed three blocks of the experimental task controlling only two VHs (each block using a different active VH combination) while the third VH was set to preserve the shape of the triangle in response to the motion of the other two VHs.

In the *training phase*, the participants were trained only in the solo configuration by conducting the main trimanual experiment mode. Each 15-minute repetition consisted of seven blocks of 32 trials (such that there were 224 trials in each training phase and 896 across the experiment). Between each block, the participants were given a 30 s break and on Day 2 there was a five-minute rest allocated between the two training phase repetitions.

### D. Assessment

Participant performance was analyzed in all training and testing phases, where the comparison between configurations was performed using the testing phase data. Since the participants were instructed to “reach for the target with the triangle's center of mass while preserving its shape”, their task performance was evaluated in terms of the following tracking performance and coordination metrics:

• The *success rate* was computed as the number of trials where the target was reached divided by the total number of trials in each block. Let *i*-th target position be denoted as $^{i}\mathbf {x}_{T}$, the CoM position be given by $\mathbf {x}_{CoM}$ and the left, right and foot-controlled VH be given by $\mathbf {x}_{L}, \mathbf {x}_{R}, \mathbf {x}_{F}$, respectively. Trial $i$ was considered successful if the participant was able to for 0.1 s hold the tracking distance $ \Vert ^{i}\mathbf {x}_{T} - \mathbf {x}_{CoM} \Vert < 0.75$ cm while maintaining all triangle lengths $\Vert \mathbf {x}_{L} - \mathbf {x}_{R} \Vert$, $\Vert \mathbf {x}_{L} - \mathbf {x}_{F} \Vert$ and $\Vert \mathbf {x}_{R} - \mathbf {x}_{F} \Vert$ within 30% of the initial length.
• The *completion time* $t_{s}$ which represented the difference of time from the target first being shown on the screen to the time that the triangle's CoM reached the target. If the target was not successfully reached, the update time was taken as the completion time.
• The *shape index* was computed as the average of the total deviation of the triangle from its initial length divided by the total time taken across the trial.
• The *coordination rate* which measured the percentage of time in which two hands were concurrently moving and keeping the edge of the triangle within 30% of the initial length in one trial.
• The *time to peak* $t_{p}$ represented the difference of time from the target first being shown on the screen to the time that the triangle's CoM reached its peak speed.

The participant's underlying motion characteristics were also evaluated to determine if those characteristics differed between the different configurations and as a result of their training. In particular, we evaluated the *motion efficiency* $\eta$ from the ratio between the shortest distance from the CoM to the target $(0\ \leq \eta \leq 1)$ and the actual travel distance.

### E. Questionnaires

Two *questionnaires* were also provided to evaluate the solo and dyad hands/foot control modes on the first and third days after each experimental condition: (i) The raw NASA TLX [19] was used to evaluate the perceived mental load including the aspects of mental, physical, temporal demands, performance, effort and frustration; (ii) The participants' ranked the tasks with respect to difficulty and their preference in a questionnaire at the end of the test on Days 1 and 3.

### F. Statistical Analysis

The Shapiro-Wilk test was applied to examine the normality of the data for each experimental condition. This found that the data was normally distributed only for the raw NASA TLX and the completion time, while all other data was found to be not-normally distributed. The training data was evaluated as a function of the training repetition, where a one-way rmANOVA was employed to investigate completion time, and the not-normally distributed success rate and motion efficiency were analysed using a Friedman test.

All comparisons considered the same participant or partner configuration as repeated measures across the two different tests. The completion time between the dyad and solo configurations on Day 1 and Day 3 were compared using a two-way rmANOVA. When analysing the not-normally distributed performance data we used a non-parametric two-way repeated measures ART rmANOVA with two factors, day {Day 1, Day 3} and the configuration condition {solo, dyad two hands, dyad foot}. Coordination improvement was assessed with ART rmANOVA with two factors, configuration and hand combination {left & right hand, left hand & foot, right hand & foot}. For rmANOVA, Maulchy's test was conducted to examine the assumption of sphericity. In case of violation of this assumption, the p-values were corrected with the Greenhouse-Geisser correction. For analysis of the ranking data (preference and difficulty), we used a Friedman test for each day separately. When the influence of the factors or their interaction had a significant effect on the participants' subjective response, we conducted post-hoc analysis with tailored comparisons. For comparisons between single conditions for the not-normally distributed metrics, post-hoc paired Wilcoxon sign-rank tests were employed, and for the normally distributed data, we conducted t-test contrasts. The Holm-Bonferroni adjustment was used to control the family-wise error rate for all conducted tests of each metric.

## Supplementary Materials

Supplementary materials show the change in the coordination and efficiency metrics over the training repetitions as well as example motion trajectories and speed profiles. As an additional metric of the participants' motion characteristics, we also investigated the portion of time in which the participants moved all virtual hands concurrently in a common direction (*parallelization rate*).

Supplementary materials
