# Supplementary material for: Can Training Make Three Arms Better Than Two Heads for Trimanual Coordination?
Source: IEEE Open J Eng Med Biol. 2023 Aug 16;4:148–55. doi: 10.1109/OJEMB.2023.3305808 (PMC10810309; doi:10.1109/OJEMB.2023.3305808)
Supplement: Supplementary materials [file supp1-3305808.pdf]

Supplementary Materials

Can training make three arms better than two heads for trimanual coordination?

Yanpei Huang, Jonathan Eden, Ekaterina Ivanova and Etienne Burdet

ADDITIONAL TRAINING METRICS

As an additional investigation of the changes of participant behaviour during solo trimanual operation, we studied the evolution of each of the testing metrics across the training repetitions. Each of these are listed below.

Motion efficiency and peak time

Fig. 1a shows the change in the motion efficiency metrics over the training sessions. Since the motion efficiency training data was found to be normally distributed, we analysed it as a function of the training session with a one-way repeated measures ANOVA (rmANOVA). The motion efficiency was observed to change with respect to session (Fig. 1a $F(3,45) = 4.71, p < 0.0061$). Here the efficiency improved between Repetitions 1 and 2 ($t(45) = -2.225, p = 0.0312$) before stabilizing between Repetitions 2 and 4 (Repetitions 2-3: $t(45) = -1.207, p = 0.2336$; Repetitions 3-4: ($t(45) = 0.390, p = 0.6981$)).

The training changes across the three days for the time to peak is shown in Fig. 1b. The time to peak was not shown to change with respect to repetition (Friedman test: $\chi^2(3) = 1.125, p = 0.771$).

Three hand coordination

To analyse how the coordination patterns between different limbs changed during the training, we used a two-way ART rmANOVA with the repetition number and the limb combination (LR: right-left, LF: left-foot, RF: right-foot) as factors. These metrics had a mixed impact of training (Fig. 1c-e, hand combination: $F(2,165) = 292.4165, p < 0.0001$; repetition number: $F(3,165) = 55.2735, p < 0.0001$; interaction: $F(6,165) = 2.5568, p = 0.0215$). Improvement occurred in the first two trials for both the LF ($Z = -3.2059, W = 6, p = 0.0039$) and RF ($Z = -2.7406, W = 15, p = 0.0334$) cases, although there was no improvement for the LR combination ($Z = -1.8615, W = 32, p = 0.3270$). This learning also did not impact the relative coordination between the hands. As expected participants were most coordinated with the LR combination (LR-LF: $Z = 3.4128, W = 134, p = 0.0010$, LR-RF: $Z = 3.5162, W = 136, p = 0.0006$). Surprisingly, though they were more coordinated in LF than RF throughout their training ($Z = 3.5162, W = 136, p = 0.0006$).

Shape index

Fig. 1f-h shows how each hand's components of the shape index changed with respect to the training repetition. The shape index was not found to change with respect to the training repetition (Friedman test: $\chi^2(3) = 1.95, p = 0.5828$).

REPRESENTATIVE TRAJECTORIES AND SPEED

Example representative motion behaviours during trials are depicted in Fig. 2. Fig. 2a illustrates the trajectories from representative participants for both Day 1 and Day 3 in the solo and dyad configuration. Here, the 32 trials for a single session are shown adjusted such that they all start at a common centre position. It can be observed that the dyad operation is similar for both Day 1 and Day 3; while solo trimanipual operation becomes slightly straighter on Day 3 compared to Day 1. Fig. 2b further illustrates the speed profiles of each hand and their center of mass using trial 15 (from the beginning of the trial to when the target was successfully reached) as an example trial for each configuration and test. From this the solo operation improvement can be observed from Day 1 to Day 3.

PARALLELIZATION METRIC

As an additional metric of the participants' motion characteristics, we investigated the portion of time in which the subjects moved all virtual hands concurrently with common direction (*parallelization rate*). This was computed as the proportion of time for which all hands had sufficiently large velocity in the direction of the centre of mass (CoM) motion. Let the CoM unit motion direction be approximated as

$$\widehat{\mathbf{p}}(t) = \frac{\dot{\mathbf{x}}_{CoM}(t)}{||\dot{\mathbf{x}}_{CoM}(t)||}, \quad \dot{\mathbf{x}}_{CoM}(t) = \frac{\mathbf{x}_{CoM}(t+1) - \mathbf{x}_{CoM}(t)}{\Delta t} \quad (1)$$

where Δt is the time step. Noting that the velocity of hand j in the CoM motion direction is given by the scalar product $\dot{p}_j = \dot{\mathbf{x}}_j \widehat{\mathbf{p}}$, the parallelization rate was then computed as

$$^{j}\rho = \frac{1}{t_s} \sum_t (\dot{p}_{j,R}(t) \geq \beta) \wedge (\dot{p}_{j,L}(t) \geq \beta) \wedge (\dot{p}_{j,S}(t) \geq \beta) \quad (2)$$

where $\beta = 0.5\,||\dot{\mathbf{x}}_{CoM}(t)||$.

Fig. 3 shows the parallelization for the different test sessions. The parallelization rate improved across the days (two-way ART rmANOVA $F(1,15) = 35.1781, p < 0.0001$), suggesting that the participants increased their concurrent motion. The dyad's motion was however more parallelized across both sessions ($F(1,15) = 42.7362, p < 0.0001$). This suggests that the naturally parallelized decision making possible from working with a dyad might still enable greater parallelization of motion within the task. It is worth noting that the final parallelization rate was relatively low at $58.98 \pm 9.01\%$ and $52.70 \pm 11.08\%$ (mean $\pm$ standard deviation) in the dyad and solo configurations, respectively. This suggests that in both configurations, the subjects still struggled to concurrently move all limbs. It is unclear if these results indicate an upper limit in trimanual coordination ability, if they are particular to the use

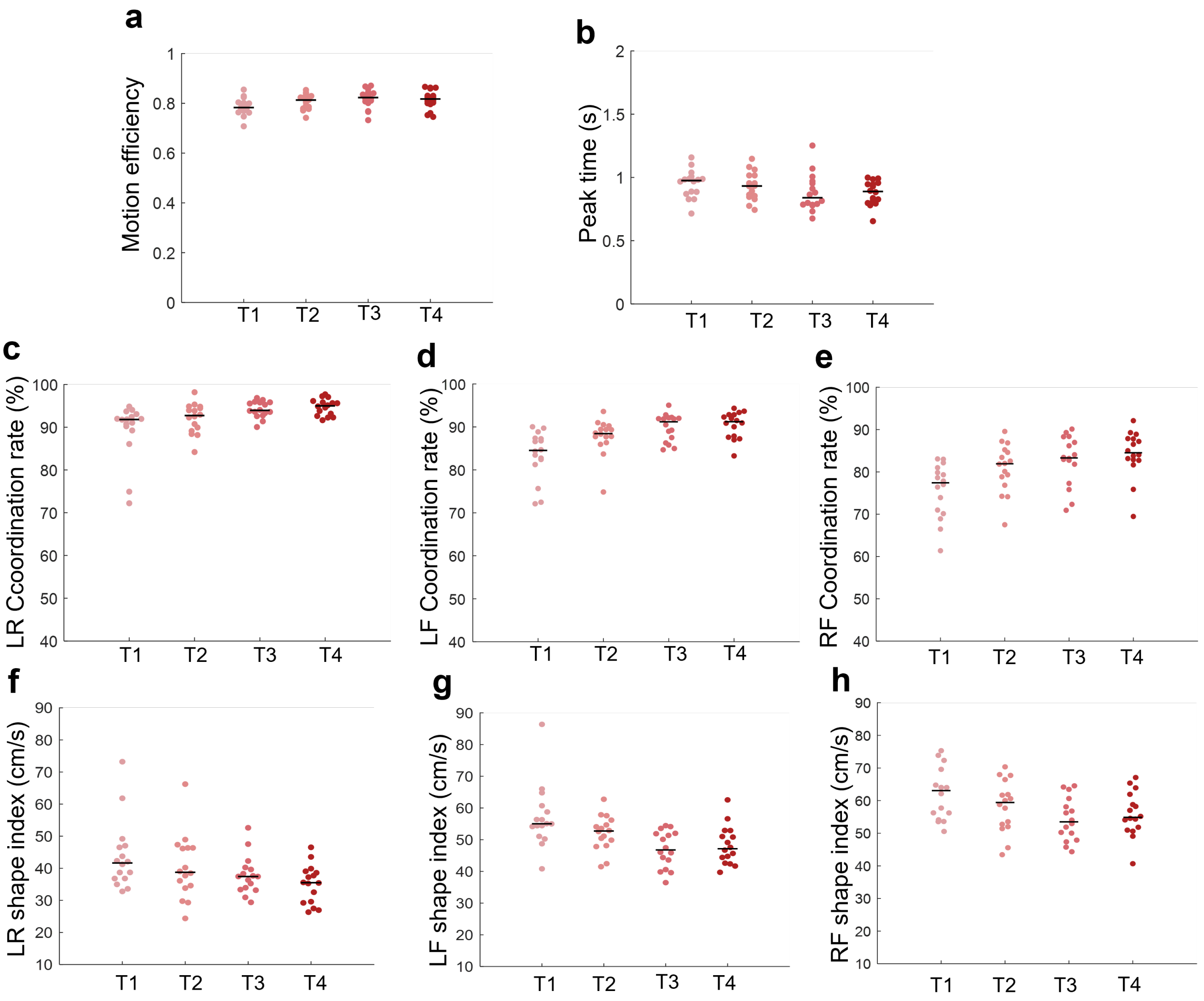

Fig. 1. Performance in training phases. The panels show first the performance of (a) Motion efficient ratio (1 is optimal) and (b) Time to peak speed. The coordination performance of every pair of virtual hands is shown in (c) left & right (LR) hand, (d) left hand & foot (LF), (e) right hand & foot (RF). The shape index of every pair of virtual hands is shown in (f) LR, (g) LF, (h) RF. T1-T4 represents Repetitions 1 to 4.

of foot-based control, or if they are unique to the chosen task where subjects may have found it easier to exploit the allowed deviation in the elastic bar then to move completely in parallel.

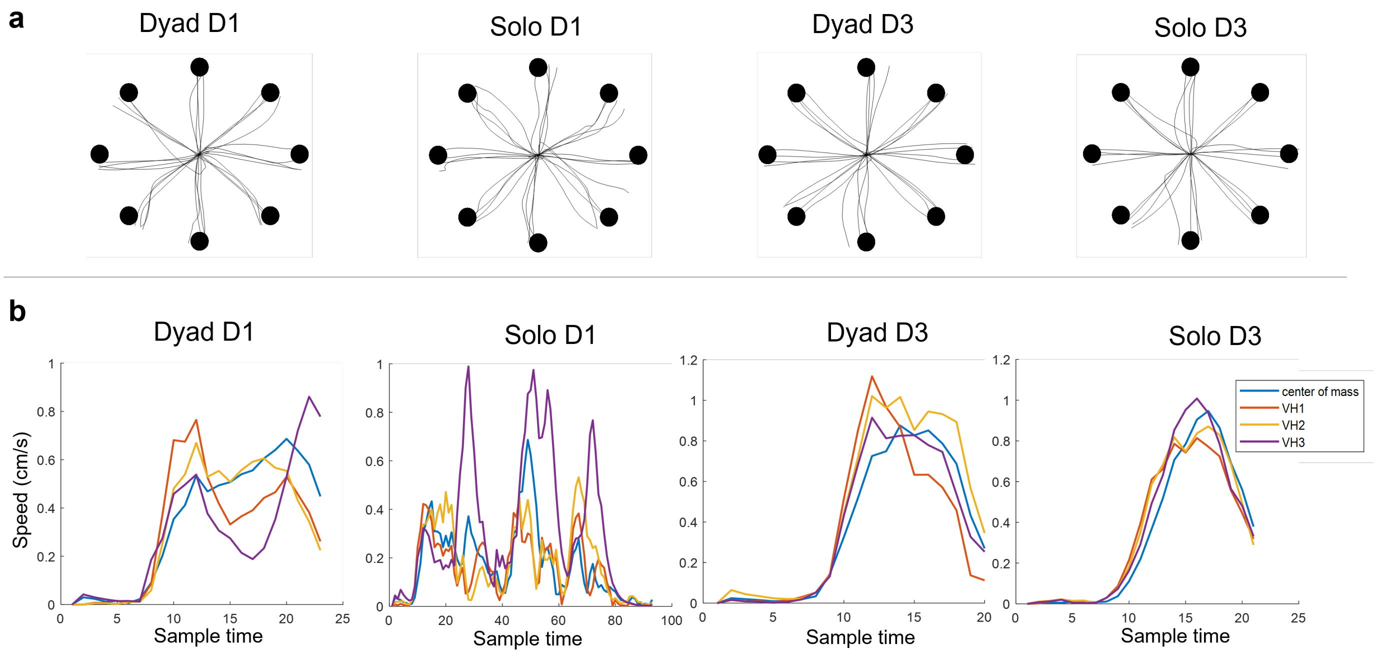

Fig. 2. Representative trajectories and speed profiles. (a) Illustration of the relative motion trajectories for a pair of representative participants for the solo and dyad configurations on Day 1 and Day 3. Note that the starting point has been offset such that each trajectory comes from a common point. (b) Example speed profiles for the same representative participants. This shows the speed of each hand and their centre of mass in trial 15 for each configuration and test.

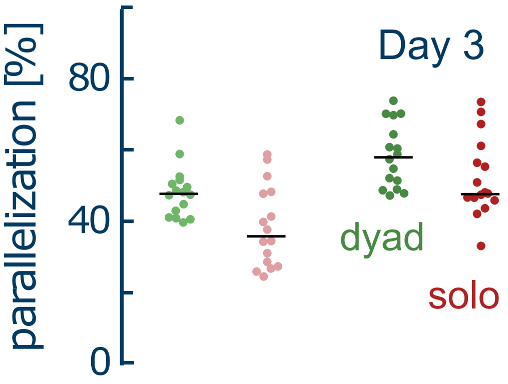

Fig. 3. Parallelization rate measure for the different configurations before and after training
